# Improved Physicochemical Properties of Yogurt Fortified with Fish Oil/γ-Oryzanol by Nanoemulsion Technology

**DOI:** 10.3390/molecules23010056

**Published:** 2018-01-02

**Authors:** Jinfeng Zhong, Rong Yang, Xiaoyi Cao, Xiong Liu, Xiaoli Qin

**Affiliations:** College of Food Science, Southwest University, Chongqing 400715, China; jfzhong@swu.edu.cn (J.Z.); YangRong6144@163.com (R.Y.); Yr931016@163.com (X.C.); liuxiong@swu.edu.cn (X.L.)

**Keywords:** fortified yogurt, physicochemical properties, nanoemulsion, fish oil, omega-3 polyunsaturated fatty acids, γ-oryzanol

## Abstract

Fish oil has several dietary benefits, but its application in food formulations is limited because of its poor water-solubility, easy oxidation and strong odor. The purposes of this study were to produce a fish oil/γ-oryzanol nanoemulsion and to evaluate the effect of adding this nanoemulsion on the physicochemical and sensory characteristics of yogurts. Adding fish oil/γ-oryzanol nanoemulsion resulted in a significant reduction in the acidity and syneresis of yogurt. Yogurt with the nanoemulsion had significantly lower peroxide value (0.28 mmol/L after 21 days) and higher retention of eicosapentaenoic acid and docosahexaenoic acid contents (decreased to 95% and 94% of its initial value, respectively) than yogurt with fish oil/γ-oryzanol (peroxide value = 0.65 mmol/L; eicosapentaenoic acid and docosahexaenoic acid contents decreased to 72% and 53% of its initial value, respectively). Fish oil/γ-oryzanol nanoemulsion incorporated into yogurt had closer sensory attributes scores to plain yogurt. This study may have important implications for the application of fish oil/γ-oryzanol nanoemulsion in yogurt.

## 1. Introduction

Omega-3 polyunsaturated fatty acids, particularly eicosapentaenoic acid (EPA) and docosahexaenoic acid (DHA), have positive health benefits, including reduction in risk of fatal coronary heart disease [1], proper development and function of human brain and prevention and treatment of many diseases [2]. Nowadays, adequate intake of fish oil in human diet is commonly recommended. A good way to increase the intake of omega-3 polyunsaturated fatty acids is to enrich regularly consumed foods with fish oil which is rich in omega-3 polyunsaturated fatty acids. Fish oil has been introduced as supplements to functional or fortified foods, but it is difficult to incorporate fish oil into most of food formulations due to its poor water-solubility which leads to its low bioavailability [3]. In addition, the easy oxidation and strong odor of fish oil also limit its use for food fortification [4]. Recent studies have developed various encapsulation technologies (e.g., emulsions, nanoemulsions and liposomes) that improve water-solubility of oils and lipophilic bioactive compounds, and most previous studies have focused on the fabrication and physical stability of the encapsulation technologies [5,6,7]. Nanoemulsion technology is one of the most effective encapsulation techniques by which lipids can be incorporated into an aqueous phase to achieve improved dispersion in the food systems, to get protection against the deterioration or degradation, and finally to enhance their bioavailability [7]. Fish oil nanoemulsion are generally prepared by high-energy methods (e.g., high pressure homogenization, sonication) [8,9]. However, the high-energy emulsification methods may impact the chemical stability of polyunsaturated fatty acids-enriched fish oil [9,10]. Promisingly, low-energy methods (e.g., spontaneous emulsification and phase inversion temperature emulsification) attract increasing interest for the development of bioactive lipids nanoemulsions including fish oil due to simple equipment, low manufacturing costs, mild conditions for nanoemulsion formation [11,12]. Most studies focused on the formation of fish oil nanoemulsions stabilized by various surfactants (or emulsifiers) using different emulsification methods and on physical and oxidative stability of fish oil nanoemulsions [13,14,15]. However, little information is available on the development and evaluation of fish oil nanoemulsions utilized in food formulations. A recent study investigated physiochemical properties (e.g., pH and acidity, syneresis, etc.,) of frozen yogurt fortified nanoliposomes containing fish oil [16]. However, still some challenges of fish oil encapsulated by nanoemulsion and its application in food products have not been solved because fish oil is relatively unstable to environmental factors (such as heat treatments, air, ionic strength) during processing and storage. 

γ-Oryzanol, a mixture of ferulic acid esters of triterpene alcohols and sterols, is one of major bioactive compounds in rice bran. γ-Oryzanol has received increasing attention due to its antioxidant activity, cholesterol-lowering and anti-inflammatory [17]. To our best knowledge, there is little information on the low-energy production of fish oil/γ-oryzanol nanoemulsion and its application in food products. γ-Oryzanol present in fish oil/γ-oryzanol nanoemulsion may act as an antioxidant protecting fish oil against deterioration and provide many health benefits. Therefore, the objectives of this study were to produce fish oil/γ-oryzanol nanoemulsion by low-energy emulsification, and to evaluate the effect of adding the nanoemulsion on the physicochemical and sensory characteristics of yogurt samples. Yogurt was chosen as a carrier system for the daily uptake of fish oil/γ-oryzanol nanoemulsion, because it is a health food product that is frequently and commonly consumed in daily diets. The addition of fish oil/γ-oryzanol nanoemulsion into yogurt may provide numerous health benefits.

## 2. Results and Discussion

### 2.1. Production of Fish Oil/γ-Oryzanol Nanoemulsion

A nanoemulsion containing 3% fish oil, 0.1% γ-oryzanol and 10% surfactants was prepared under the given conditions, and had a mean particle size of about 152 nm and polydispersity index of 0.23 (Figure S1). The fish oil/γ-oryzanol nanoemulsion stabilized by Tween 80/Span 20 was physically stable at a broad pH range (2–7) and at 5 °C for 30 days [18], which is good for its application in yogurt products. For emulsions made with protein as an emulsifier, they were unstable under acid conditions (i.e., isoelectric point of the protein) [19], which limits their application in acid food products like yogurt and beverage.

### 2.2. Effect of Adding Fish Oil/γ-Oryzanol Nanoemulsion on Physicochemical Properties of Yogurt

#### 2.2.1. pH and Acidity

**Figure 1:**
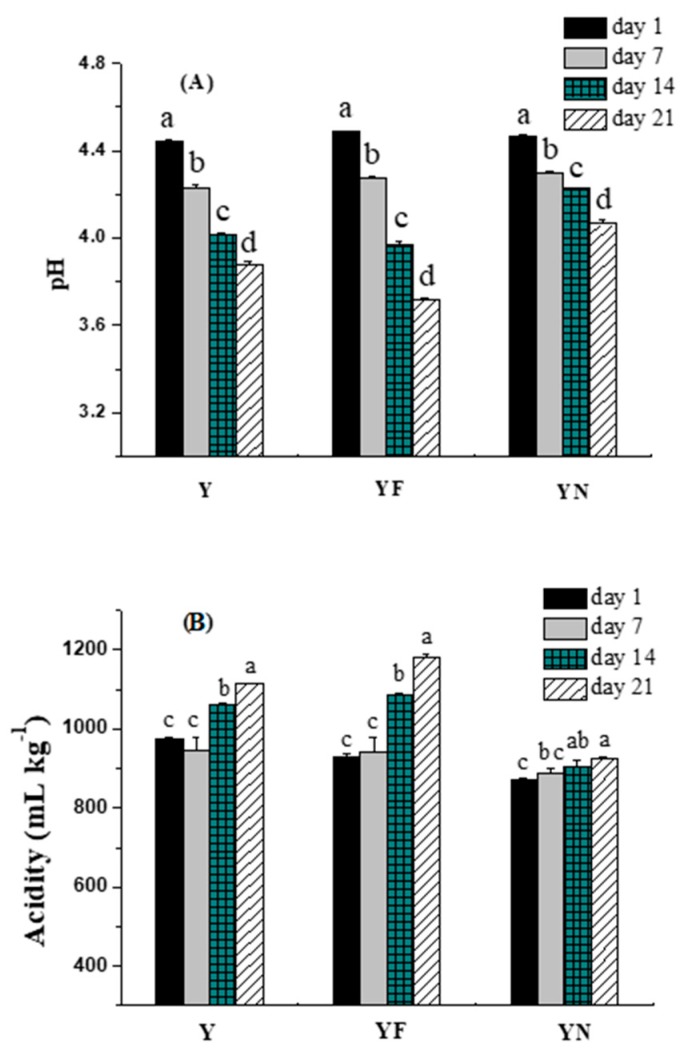
Changes in pH (**A**) and acidity (**B**) of yogurt samples during 21 days storage at 4 °C. Y: plain yogurt; YF: yogurt fortified with fish oil/γ-oryzanol; YN: yogurt fortified with fish oil/γ-oryzanol nanoemulsion. Values affected by storage time having different lower case letters are significantly different (*p* < 0.05).

pH and acidity are important to the taste of yogurt. The pH and acidity of yogurt within shelf-life are generally 4.0–4.6 and 70–110 mL NaOH/kg yogurt, respectively [20]. Figure 1 shows changes in pH and acidity of different yogurt samples during 21 days of storage at 4 °C. Generally, pH decreased in control yogurt over time, which was probably due to the production of lactic acid by starter culture bacteria. As seen in Figure 1A, the sharpest decline of pH was found in positive control sample (yogurt fortified with fish oil/γ-oryzanol). Acidity in the positive control sample was more dramatically increase after 14 days storage (Figure 1B). In addition to the accumulation of lactic acid, the hydrolysis and oxidation of oils may also contribute to the sharpest decline of pH and increase of acidity in the positive control. Interestingly, yogurt fortified with fish oil/γ-oryzanol nanoemulsion could attenuate the decline of pH and increase of acidity. Oils present in the encapsulation form in the nanoemulsion were more stable to hydrolysis and oxidation, leading to less postacidification of yogurt fortified with fish oil/γ-oryzanol nanoemulsion during storage (Figure 1B). The possible reason is that oil droplets in the nanoemulsion are covered by a layer of the surfactants which can reduce chemical reactions between the oil and a number of water and air. Similar results were observed by Ghorbanzade et al. [16] who reported that higher acidity and lower pH were obtained in the plain yogurt than in yogurt fortified with fish oil, but there was small differences in acidity and pH between yogurt containing fish oil and yogurt containing nano-liposomal encapsulated fish oil. In this study, yogurt fortified with fish oil/γ-oryzanol nanoemulsion displayed better values of pH and acidity over 21 days of storage than the plain yogurt and yogurt containing fish oil.

#### 2.2.2. Viscosity of Yogurts

**Figure 2:**
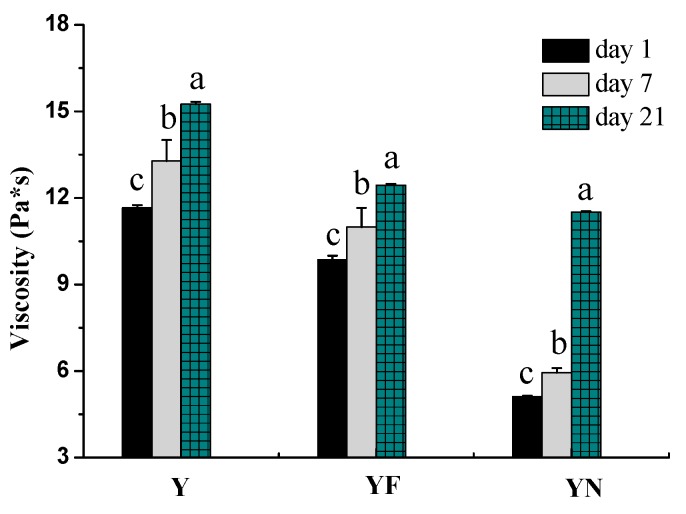
Viscosity changes in yogurt samples during 21 days storage at 4 °C. Y: plain yogurt; YF: yogurt fortified with fish oil/γ-oryzanol; YN: yogurt fortified with fish oil/γ-oryzanol nanoemulsion. Values affected by storage time having different lower case letters are significantly different (*p* < 0.05).

Figure 2 shows changes in apparent viscosity of yogurt samples during storage period. Apparent viscosity values increased throughout the storage period, which is in agreement with recent reports in the literature [21,22]. The addition of fish oil/γ-oryzanol nanoemulsion significantly decreased the apparent viscosity of fortified yogurt compared to negative sample (plain yogurt). The significant decrease in apparent viscosity at day 1 can be attributed to the dilution of fortified yogurt by the addition of fish oil/γ-oryzanol nanoemulsion containing a lot of water. On the other hand, set-style yogurt used in this study is a pseudo-plastic fluid, and the apparent viscosity was decreased when the fish oil/γ-oryzanol nanoemulsion was thoroughly mixed with the yogurt. However, apparent viscosity values of yogurt fortified with fish oil/γ-oryzanol nanoemulsion increased with storage time, and the degree of increase in viscosity value varied more markedly than that in the other two groups, which may cause by the reason that large quantities of water molecules in nanoemulsion hydrated protein present in yogurt with storage time. 

#### 2.2.3. Syneresis of Yogurt Samples

**Figure 3:**
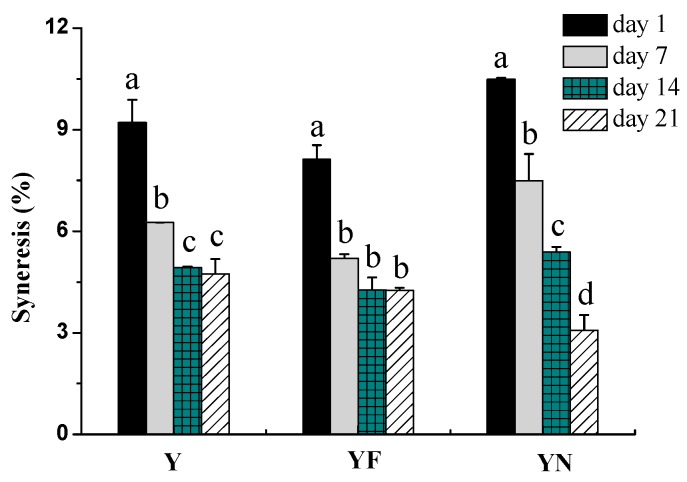
Syneresis changes in yogurt samples during 21 days storage at 4 °C. Y: plain yogurt; YF: yogurt fortified with fish oil/γ-oryzanol; YN: yogurt fortified with fish oil/γ-oryzanol nanoemulsion. Values affected by storage time having different lower case letters are significantly different (*p* < 0.05).

Syneresis is another important factor assessing the quality of yogurt. Figure 3 shows effect of the form of fish oil (free form or incorporated in nanoemulsion) on syneresis in yogurt samples during 21 days storage. The amount of syneresis decreased with increasing storage time, and the highest syneresis was achieved during the first day of storage. This could be explained that pH (about 4.5) of yogurt samples during the first day of storage (Figure 1A) was close to the isoelectric point of casein present in yogurt samples, leading to the yogurt system having a lower water-holding capacity. Besides, gel structure of yogurt samples was broken before storage due to gently mixing of fish oil/γ-oryzanol or fish oil/γ-oryzanol nanoemulsion into yogurt sample, which may also contributed to a much higher syneresis in the first day of storage. Compared to control groups, the fish oil/γ-oryzanol nanoemulsion-fortified yogurt had the least syneresis during the last week of storage, although it yielded the highest syneresis during the first week (Figure 3) and lower apparent viscosity during the last week of storage (Figure 2). This result may contribute to improved water-holding capacity of yogurt system by the presence of surfactants in the yogurt fortified with the fish oil/γ-oryzanol nanoemulsion. Previous publication reported that syneresis of yogurts also could be reduced by increasing the density of matrix or the proportion of solids contents by adding protein [23], inulin and maltodextrin [24], etc.

#### 2.2.4. Fatty Acid Composition of Yogurts

**Figure 4:**
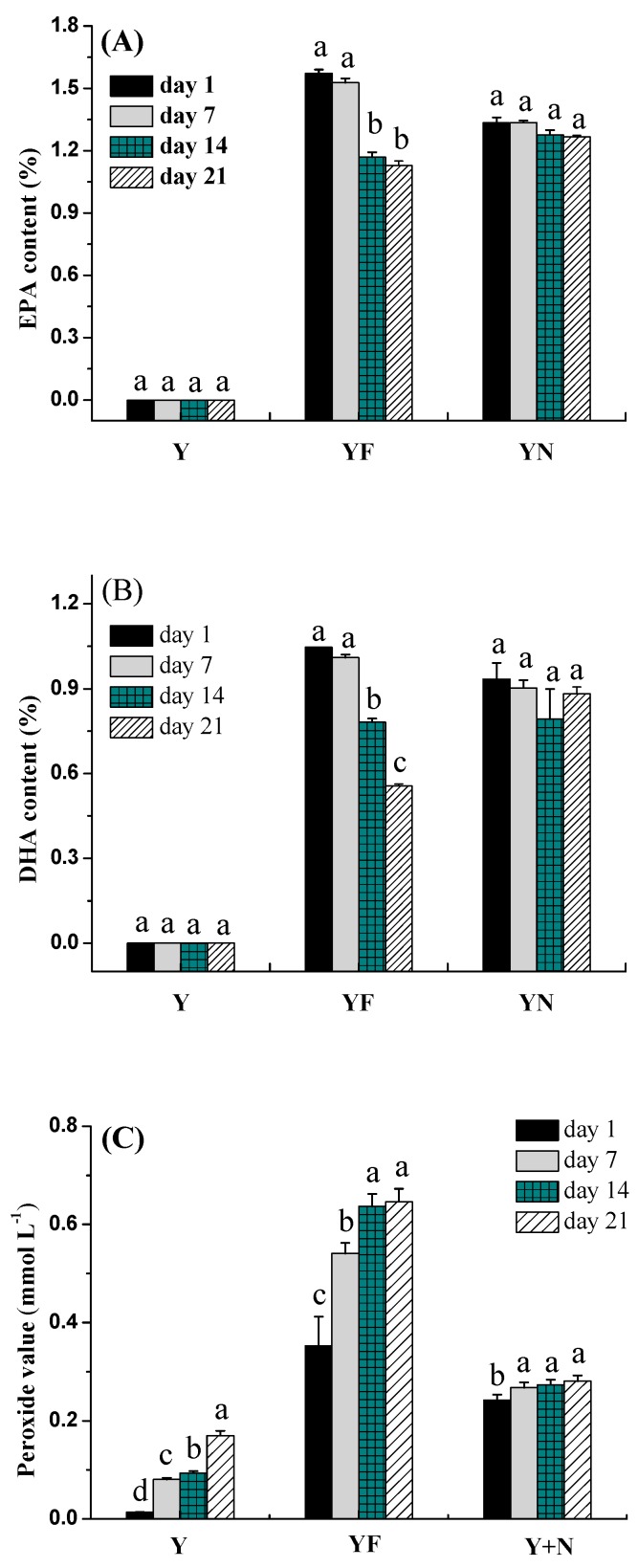
Fatty acid composition (**A**) and peroxide value changes (**B**,**C**) in yogurt samples during 21 days storage at 4 °C. Y: plain yogurt; YF: yogurt fortified with fish oil/γ-oryzanol; YN: yogurt fortified with fish oil/γ-oryzanol nanoemulsion. Values affected by storage time having different lower case letters are significantly different (*p* < 0.05).

Changes in fatty acid composition can evaluate the chemical stability of oil in various formulations. Figure 4A,B display the changes in fatty acid composition of different treatments of yogurt samples during 21 days of storage. Generally, plain yogurt does not contain polyunsaturated fatty acids, and it mainly contains monounsaturated fatty acid (oleic acid) and saturated fatty acids (palmitic acid, myristic acid, lauric acid). The addition of fish oil (incorporated in nanoemulsion or free form) can achieve fortified yogurt containing omega-3 polyunsaturated fatty acids such as EPA and DHA (Figure 4A,B). 

EPA and DHA contents decreased with increasing storage time. A much remarkable decrease was observed especially during the last two weeks of storage for yogurt fortified with fish oil/γ-oryzanol. EPA content and DHA content of the yogurt fortified with fish oil/γ-oryzanol decreased to about 72% and 53% of its initial value after 21 days, respectively. The significant reduction in EPA and DHA contents indicated omega-3 polyunsaturated fatty acids were susceptibly oxidized in yogurt fortified with fish oil/γ-oryzanol. However, the yogurt fortified with fish oil/γ-oryzanol nanoemulsion had greater retention of EPA and DHA after 21 days (about 95% and 94% of its initial value, respectively). The reason could be probably due to better protection of EPA and DHA against environmental deterioration factors by the nanoemulsion. Similarly, Ghorbanzade et al. [16] reported that polyunsaturated fatty acids could be effectively protected by nano-liposome, EPA content and DHA content of yogurt fortified with unencapsulated fish oil decreased to 50% of its initial value after 21 days of storage.

#### 2.2.5. Peroxide Value of Yogurts

Peroxide value was used to evaluate the primary oxidation of yogurt containing oil. As shown in Figure 4C, the peroxide value of plain yogurt was less than 0.17 mmol/L after 21 days of storage, since the milk fat in yogurt contained more saturated fatty acids which are relatively stable to oxidation. Much more severe oxidation occurred in yogurt fortified with fish oil/γ-oryzanol. Peroxide value of yogurt fortified with fish oil/γ-oryzanol was 0.65 mmol/L after 21 days storage which was 1.8 times of that in the first day. Interestingly, a very slow increase in peroxide value was observed for yogurt fortified with fish oil/γ-oryzanol nanoemulsion, and peroxide value increased from 0.24 mmol/L in the first day to 0.28 mmol/L after 21 days storage. These results further confirmed that nanoemulsion as one of technologies for encapsulating fish oil could effectively protected polyunsaturated fatty acids against deterioration by oxidation. 

#### 2.2.6. Melting and Crystallization Behavior of Yogurts

Table 1 shows overall enthalpy (△H), extrapolated onset and endothermic peak temperatures of different yogurt samples. Overall enthalpy (△H) of yogurt samples with different treatments were non-significantly different (*p* > 0.05), indicating that similar heat energy was required for melting the yogurt samples. There was no significant difference (*p* > 0.05) in extrapolated onset temperature which the yogurt starting melting between different yogurt samples. Similar results were obtained for endothermic peak temperature which around 0 °C. An endothermic peak appeared at the temperature suggested it corresponded to melting of yogurt. DSC data showed that the addition of fish oil/γ-oryzanol nanoemulsion or fish oil/γ-oryzanol did not affect the melting point of yogurt mixes.

### 2.3. Sensory Evaluation of Yogurts

Table 2 lists scores of sensory evaluation of yogurt samples stored in the 1st day and the 15th day. Plain yogurt scored better color than fortified yogurts. In terms of taste and aroma, yogurt fortified with fish oil/γ-oryzanol had the lowest score, followed by yogurt fortified with fish oil/γ-oryzanol nanoemulsion and plain yogurt, suggesting fish oil/γ-oryzanol incorporated in nanoemulsion particles could attenuate taste and aroma of yogurt. Texture of yogurt samples was also improved by the addition of fish oil/γ-oryzanol nanoemulsion rather than by the addition of fish oil/γ-oryzanol. Sensory data showed that sensory scores of yogurt fortified with fish oil/γ-oryzanol nanoemulsion were higher than yogurt fortified with fish oil/γ-oryzanol, and yogurt fortified with fish oil/γ-oryzanol nanoemulsion was more acceptable to consumers than yogurt fortified with fish oil/γ-oryzanol. Fish oil has a low flavor threshold, level of fish oil and food systems could affect flavor or aroma in fish oil-containing foods. It was found that a significant difference (*p* < 0.05) was in sensory perception was detected when the level of fish oil addition was more than 4% in cheese fortified with fish oil emulsion stored for 1 week [25]. Compared to yogurt fortified with fish oil/γ-oryzanol, yogurt fortified with fish oil nanoliposome gave closer score of sensory attributes to plain yogurt when the level of fish oil was very low (about 0.26%) in the yogurt [16]. If the addition amount of fish oil (incorporated in nanoliposome) is at higher levels like 1–4% in yogurt, it is unknown that whether the sensory score of yogurt fortified with fish oil nano-liposome was significantly lower than that of the plain yogurt. In this study, the addition amount of fish oil (incorporated in nanoemulsion or free form) in yogurt was high (3%), which meets recommended daily intake of omega-3 polyunsaturated fatty acids. In addition, a plain yogurt was used as a carrier to evaluate the effect of form of fish oil (incorporated in nanoemulsion or free form) on sensory characteristics of yogurt. The nanoemulsion could attenuate fishy odor, but the sensory score of yogurt fortified with fish oil/γ-oryzanol nanoemulsion was a little lower than that of plain yogurt. It is possible that the use of flavor yogurt as a carrier and moderate decrease in fish oil concentration will increase the the acceptability (taste) of yogurt fortified with fish oil/γ-oryzanol nanoemulsion in future research. 

## 3. Materials and Methods

### 3.1. Materials

Fish oil (EPA 19.6%, DHA 14.0%) was kindly donated by Sinomega Biotech Engineering Co., Ltd. (Zhoushan, China). γ-Oryzanol (purity > 98%) was purchased from Dalian Meilun Biotech Co., Ltd. (Dalian, China). Yogurt (freshly made) was purchased from Chongqing Tianyou Dairy Co., Ltd. (Chongqing, China), and the composition of the yogurt was 3.2% protein, 3.5% fat and 10% carbohydrate. Medium-chain triglyceride (MCT) was purchased from Shanghai Yiji Industrial Co., Ltd. (Shanghai, China). All other chemicals used were of analytical grade and obtained commercially.

### 3.2. Production of Fish Oil/γ-Oryzanol Nanoemulsion and Its Particle Size Measurement

A nanoemulsion technique was use to encapsulate fish oil and γ-oryzanol. It was made by low-energy emulsification by injecting an organic phase into an aqueous phase, according to our previously study [18]. Initially, 0.1 g γ-oryzanol dissolved in 10 g oil phase (fish oil/MCT weight ratio = 3:7) and 10 g mixed surfactants (Tween 80/Span 20 weight ratio = 3:1) were stirred at 850 r/min at least 20 min at room temperature to obtain well mixed organic phase. Aqueous phase (80 g) is a deionized water solution that consisted of 1% citric acid and 0.1% sodium benzoate. Then, the well mixed organic phase was added to the aqueous phase under stirring (850 r/min) by a dropping funnel. The titrating speed of the organic phase was hold at 60 drop/min. An additional 5 min was allowed for stirring. 

The particle size distribution of the resultant fish oil/γ-oryzanol nanoemulsion was determined using the Malvern Zetasizer Nano ZS90 (Malvern Instruments, Worcestershire, UK). To prevent multiple scattering, the fish oil/γ-oryzanol nanoemulsion sample was diluted 500 times with deionized water buffer containing 1% citric acid and 0.1% sodium benzoate. Each recorded measurement was an average of 13 runs. Samples were measured at least in duplicate at 25 °C. The results of particle size are presented as the z-average particle diameter.

### 3.3. Preparation of Yogurt Fortified with Fish Oil/γ-Oryzanol Nanoemulsion 

The formulation of yogurt fortified with fish oil/γ-oryzanol nanoemulsion was made by adding 13 g fish oil/γ-oryzanol nanoemulsion into 100 g yogurt sample and stored in a tightly closed glass container in a refrigerator at 4 °C for 21 days. Similar procedures were carried out for two control yogurt samples. Plain yogurt was as negative control. Yogurt containing the oil phase and γ-oryzanol was as positive control which formulation was made by adding 1.3 g the oil phase and 12.9 mg γ-oryzanol into 100 g yogurt sample. Both the positive control and yogurt fortified with the fish oil/γ-oryzanol nanoemulsion contained the same level of γ-oryzanol and the oil phase. All yogurt samples were prepared in duplicated. Yogurt samples were taken out periodically for further analysis. 

### 3.4. Physicochemical Properties of Yogurt Samples

#### 3.4.1. Acidity and pH

Titrable acidity was determined according to the National Standards of the People’s Republic of China [26]. Briefly, 10 g of yogurt sample was mixed with 20 mL of boiled deionized water and titrating with 0.1 mol/L NaOH to pH 8.3. The titrable acidity was expressed as mL NaOH/kg yogurt sample. The pH of yogurt sample was measured by mixing 10 g of yogurt sample with 20 mL of boiled deionized water and recording by PHS-3C pH meter (Shanghai Electronics Science Instrument Co., Ltd., Shanghai, China). 

#### 3.4.2. Syneresis

Syneresis of yogurt samples was measured by placing yogurt sample (15 g) in the glass funnel with 200 mesh cloth at room temperature for 90 min. The separated liquid was collected in a beaker. Syneresis percentage was calculated by Equation (1):

(1) $$\mathrm{Syneresis}(\%)=\frac{\mathrm{Total}\;\mathrm{weight}\;\mathrm{of}\;\mathrm{seperated}\;\mathrm{liqiud}(g)}{\mathrm{Total}\;\mathrm{weight}\;\mathrm{of}\;\mathrm{yogurt}(g)}\times 100$$

#### 3.4.3. Viscosity Property

Viscosity of yogurt sample was determined using an NDJ-8S digital rotary viscometer (Shanghai Sunny Hengping Scientific Instrument Co., Ltd., Shanghai, China). The procedure was performed according to its operation manual.

#### 3.4.4. Chemical Stability Testing

Chemical stability of yogurt samples was evaluated by determining changes in primary lipid oxidation products and fatty acid composition. Lipid hydroperoxide (primary products) content of yogurt samples, expressed as peroxide value, was determined by using a method of Shantha and Decker [27] with slight modification. Briefly, each yogurt sample (3 mL) was mixed with 8 mL of isooctane/2-propanol (3:1, *v*/*v*) and vortexed four times for 10 s each. After centrifugation at 10,000 r/min for 15 min (Centrifuge 5804R, Eppendorf, Hamburg, Germany), the upper layer (1.6 mL) was added to 8.4 mL of methanol/butanol (2:1, *v*/*v*) followed by 45 μL of 3.94 mol/L ammonium thiocyanate and 45 μL of Fe^2+^ solution (fresh made). After 10 s of vortex and 20 min of incubation at room temperature in the dark, the absorbance of the solution was measured at 510 nm (against isooctane) by the UV-2450 UV-visible spectrophometer (Shimadzu, Kyoto, Japan). A 1.6 mL of isooctane/2-propanol (3:1, *v*/*v*) instead of the extracts from yogurt sample served as the negative control. Peroxide value was determined using a standard curve prepared from Fe^3+^ (0–16 μg/mL, *y* = 0.221*x* + 0.0174, *R*^2^ = 0.9971), and calculated using the Equation (2):

(2) $$\mathrm{PV}=\frac{(A-b)\times 8\times 10.09}{a\times 3\times 2\times 56\times 1.6}$$

where PV represents peroxide value of a yogurt sample (mmol hydroperoxide/L yogurt sample); A is absorbance of a yogurt sample against isooctane and negative control; a and b are the slope and intercept of the standard curve, respectively; 56 is the molar mass of Fe^3+^ (g/mol); 2 is the molar ratio of Fe^3+^ to hydroperoxide; 10.09 is the volume of sample reaction (mL); 3 is the volume of yogurt sample.

Fatty acid composition was used to evaluate chemical stability of fat in yogurt samples during 21 days storage. First, to extracted fat, 2.4 mL of yogurt sample was well mixed with 1.6 mL of deionized water, 10 mL of methanol and 4.4 mL of chloroform. Then, the mixture was diluted with 5 mL of deionized water and 5 mL of chloroform. After vortexing for 2 min and centrifugation (10,000 r/min, 15 min), the under layer was transferred to a glass tube and concentrated to about 1 mL under nitrogen. The concentrated extracts containing oil was then transferred to a 1.5 mL tube with the addition of anhydrous sodium sulfate. After vortexing and centrifugation (10,000 r/min, 3 min), the supernatant was transferred to a glass tube for methylation. Fifteen μL of the supernatant containing oil was added to a test tube with stopper, and 2 mL of hexane was added to the test tube for dissolving the oil. The hexane solution was methylated with 2 mL of KOH (0.5 mol/L) in methanol, vortexing for 5 min at 25 °C. Five mL of saturated sodium chloride solution was then added to the test tube. After mixing for 10 s and centrifugation (4000 r/min, 3 min), the upper layer was transferred to a glass vial for gas chromatograph (GC) analysis. The upper layer containing fatty acid methyl esters was determined using a GC (GC-2010, Shimadzu, Tokyo, Japan) equipped with a flame ionization detector and a capillary column DB-23 (60 m × 0.25 mm × 0.25 μm) (Agilent Technologies, Santa Clara, CA, USA). Injector and detector temperatures were set as 250 and 280 °C, respectively. Each sample was injected manually (1 μL) at a carrier gas (nitrogen) flow of 1 mL/min with split ratio of 1:30. The oven temperature rose from 60 to 220 °C at a rate of 7 °C/min. Fatty acid data were collected and analyzed by GC Solution (Shimadzu), and presented as weight percent of the total fatty acids.

#### 3.4.5. Freezing and Melting Behavior of Yogurts

Freezing and melting behavior of various yogurt samples was measured by a differential scanning calorimeter (DSC, DSC 4000, Perkin Elmer, Waltham, MA, USA). Yogurt sample (10–15 mg) was weighted into a stainless steel sample pan and sealed with a press. The DSC experimental program was as follows: cooling to −50 °C, holding isothermally for 5 min and then heating to 50 °C at a rate of 5 °C/min, holding isothermally for 5 min, according to the method of Alfaro et al. [28]. 

### 3.5. Sensory Evaluation

Sensory evaluation of yogurt samples including color, texture, taste and aroma, and overall acceptance was carried out by 20 trained panelists aged between 20 and 30 years old. The evaluation was done by using 5-point hedonic scales (1 = dislike extremely, 5 = like extremely). Yogurt samples placed in cups which were randomly coded with 3 digit number were present to the panelists and scored individually at room temperature. In order to ensure accurate data collection, panelists had no verbal communication with each other during the evaluation process. The sample size was large enough for the panelists to re-taste the yogurts if they desired. Water was also provided for the panelists to rinse their mouths between samples.

### 3.6. Statistical Analysis

All experiments were carried out at least in duplicate and all measurements were performed at least in triplicate. Results were present as mean values ± standard deviation. Statistical analysis of data was performed using the SPSS 18.0 statistical analysis software (Demo version; Armonk, NY, USA). Significant differences between observed mean values were by analysis of variance. Statistical significance was taken at *p* < 0.05.

## 4. Conclusions

Fish oil and γ-oryzanol were successfully incorporated as nutraceuticals into nanoemulsions. Adding this nanoemulsion containing fish oil and γ-oryzanol into yogurt resulted in a significant reduction in acidity, syneresis and peroxide value with maximum retention of EPA and DHA. Yogurt fortified with fish oil/γ-oryzanol nanoemulsion had lower viscosity than control yogurt samples, but had acceptable values. The addition of the nanoemulsion did not affect the melting and crystallization behavior of fortified yogurt mixes. Sensory evaluation data showed yogurt fortified with fish oil/γ-oryzanol nanoemulsion possessed closer scores of sensory attributes to plain yogurt than yogurt fortified with fish oil and γ-oryzanol. This study demonstrated that yogurt could be fortified with fish oil/γ-oryzanol nanoemulsion to create a potential product with considerable dietary benefits. Further studies should be carried out to improve taste and aroma of yogurt fortified with nanoemulsion containing fish oil and γ-oryzanol.

## Figures and Tables

**Table 1:** Differential scanning calorimetry analysis for different yogurt samples.

Sample ^†^	Overall Enthalpy (J g^−1^)	Extrapolated Onset Temperature (°C)	Endothermic Peak Temperature (°C)
Y-day 1	257.69 ± 28.55 ^a^	−2.72 ± 0.08 ^a^	3.58 ± 0.01 ^a^
YF-day 1	349.55 ± 29.47 ^a^	−2.73 ± 0.24 ^a^	3.62 ± 0.45 ^a^
YN-day 1	245.42 ± 17.35 ^a^	−2.35 ± 0.14 ^a^	3.31 ± 0.23 ^a^

^a^ Values in the same column having the same letter are not significantly different (*p* > 0.05). ^†^ Y-day 1: plain yogurt stored in the 1st day; YF-day 1: yogurt fortified with fish oil/γ-oryzanol stored in the 1st day; YN-day 1: yogurt fortified with fish oil/γ-oryzanol nanoemulsion stored in the 1st day.

**Table 2:** Effect of the form of fish oil on the sensory characteristics of yogurt samples.

Yogurt Samples ^†^	Sensory Quality Score			
	Color	Taste and Aroma	Texture	Overall Acceptance
Y-day 1	4.7 ± 0.5 ^a^	4.5 ± 0.5 ^a^	4.5 ± 0.5 ^a^	4.6 ± 0.5 ^a^
YF-day 1	4.1 ± 0.3 ^b^	1.3 ± 0.5 ^c^	3.7 ± 0.5 ^c^	2.2 ± 0.5 ^c^
YN-day 1	4.3 ± 0.4 ^b^	2.7 ± 0.5 ^b^	4.1 ± 0.4 ^b^	3.5 ± 0.6 ^b^
Y-day 15	4.9 ± 0.3 ^a^	4.8 ± 0.4 ^a^	3.7 ± 0.7 ^a^	4.4 ± 0.5 ^a^
YF-day 15	4.1 ± 0.2 ^b^	1.4 ± 0.6 ^c^	3.6 ± 0.6 ^a^	2.1 ± 0.6 ^b^
YN-day 15	4.2 ± 0.4 ^b^	2.1 ± 0.6 ^b^	3.8 ± 0.4 ^a^	3.2 ± 0.5 ^c^

^a–c^ Values in the same column (at the same day) having different low-case letters are significantly different (*p* < 0.05). ^†^ Y-day 1 and Y-day 15: plain yogurt stored in the 1st day and 15th day, respectively; YF-day 1 and YF-day 15: yogurt fortified with fish oil/γ-oryzanol stored in the 1st day and 15th day, respectively; YN-day 1 and YN-day 15: yogurt fortified with fish oil/γ-oryzanol nanoemulsion stored in the 1st day and 15th day, respectively.

## Supplementary Materials

The following are available online, Figure S1: Particle size distribution of fish oil/γ-oryzanol nanoemulsion.
