# Supplementary material for: Improved Physicochemical Properties of Yogurt Fortified with Fish Oil/γ-Oryzanol by Nanoemulsion Technology
Source: Molecules. 2018 Jan 2;23(1):56. doi: 10.3390/molecules23010056 (PMC6017217; doi:10.3390/molecules23010056)
Supplement: Supplementary file 1 [file molecules-23-00056-s001.pdf]

Improved Physicochemical Properties of Yogurt Fortified with Fish Oil/γ-oryzanol by Nanoemulsion Technology

Jinfeng Zhong, Rong Yang, Xiaoyi Cao, Xiong Liu and Xiaoli Qin*

College of Food Science, Southwest University, Chongqing 400715, P. R. China
* Correspondence: qinxl@swu.edu.cn; Tel.&Fax:+86-023-68251298

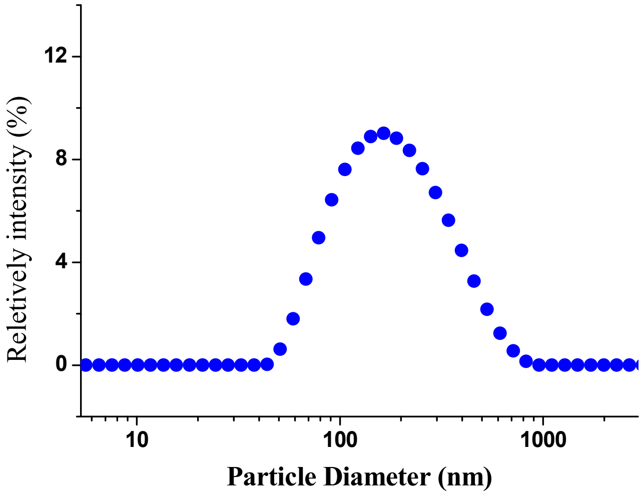

Figure. S1 Particle size distribution of fish oil/γ-oryzanol nanoemulsion
